# Matrix Metalloproteinase-1 Gene Polymorphism Associated with Ultrasound-Assessed Carotid Thickness among Older Adults

**DOI:** 10.1155/2018/1475890

**Published:** 2018-06-21

**Authors:** Gilberto Santos Morais Junior, Nathalia Oliveira Rodrigues, Adriane Dallanora Henriques, Audrey Cecília Tonet-Furioso, Ciro José Brito, Lucy Oliveira Gomes, Clayton Franco Moraes, Otávio Toledo Nóbrega

**Affiliations:** ^1^Universidade de Brasília, 70910-900 Brasília, DF, Brazil; ^2^Universidade Católica de Brasília, 71966-700 Águas Claras, DF, Brazil; ^3^Universidade Federal de Juiz de Fora, 36036-330 Juiz de Fora, MG, Brazil

## Abstract

**Background and Aim:**

Due to the high incidence of vascular diseases, it is necessary to identify new circulating or structural markers for predicting risk for chronic diseases. Some studies suggest that MMP1 gene polymorphisms are associated with the enzyme expression levels in situ (e.g., in atherosclerotic plaques).

**Objectives:**

Thus, the study of this polymorphism may help understanding the pathophysiology of coronary disease.

**Methods:**

We performed cross-sectional clinical and laboratory evaluations (including measurement of intima-media thickness of carotid arteries) and genotyping of the MMP1 SNP rs495366 (A/G) in 366 elderly people.

**Results:**

No significant differences between genotypes were noted for biochemical, metabolic, inflammatory, or clinical variables except for a significant difference in intima-media thickness for the left carotid artery and a trend toward significance for the right counterpart.

**Conclusion:**

Carriers of the allele associated with lower MMP1 expression (allele A) presented greater carotid thickness. We suggest that the phenomenon can be explained by impaired remodeling of the arterial wall (poor degradation of collagen fibers in this scenario), yielding carotid wall thickening and a greater intrinsic risk for cerebrovascular events.

## 1. Introduction

The World Health Organization (WHO) reports that 60% of deaths worldwide are due to chronic diseases, mainly vascular-related disorders [1], and estimates that this value will reach 80% in developing countries by 2020 [2]. In Brazil, approximately one-third of the deaths of individuals over 60 years of age are caused by vascular events, a proportion similar to that in the Federal District [3]. Circulatory disorders associate with clinical and biological findings such as obesity, systemic arterial hypertension, type 2 diabetes mellitus, dyslipidemias, and genetic factors that, altogether, interact and act as risk factors to the onset and progression of vascular diseases and consequently with the mortality of affected patients. In this context, atherosclerosis stands out as an underlying cause, as it triggers the highest proportion of fatal or disabling events [4].

Atherosclerosis results from chronic inflammation of the arterial wall triggered in response to endogenously modified structures, particularly oxidized lipoproteins. It is widely recognized that stimulation by circulating mediators of innate and adaptive immune responses leads to further vascular wall damage, promoting disease progression [5] with evidence that the imbalance between pro- and anti-inflammatory factors plays a critical role in early vascular endothelial dysfunction [6].

Atherosclerosis is a progressive and irreversible process resulting from a series of specific cellular and molecular factors that affect large and medium elastic or muscular arteries [7]. The main concern is the possibility of causing thrombosis or severe arterial stenosis, leading to life-threatening adverse events, such as myocardial infarction, stroke, and ischemic injuries of the kidneys and intestines, as well as other manifestations. Avoiding morbidity and mortality from circulatory causes justifies the significant interest in the identification of asymptomatic patients at risk. In this context, the thickening of the intima-media layer of the common carotid artery is a subclinical biomarker that is easy to measure with reasonable predictive capacity for relevant vascular events [8]. There is a body of evidence in the literature that associates measurements of carotid intima-media thickness (cIMT) with cardiovascular risk [9–11]. On what concerns the cerebrovascular setting, Eigenbrodt et al. [12] and Baldassarre et al. [13] independently presented evidence for an association between carotid thickness and stroke events, suggesting that this measure can be a valuable prognostic biomarker.

The development of a vulnerable atherosclerotic plaque involves intense inflammation in the presence of lymphocytes, macrophages, and mast cells. In this milieu, matrix metalloproteinases (MMPs) are typically secreted by macrophages that progressively degrade the collagenous components of the fibrous capsule. MMPs are endopeptidases that contain zinc and/or calcium within their active sites and degrade extracellular matrix proteins, such as collagen fibers, thus compromising the integrity of the endothelial basement membrane and plaque stability with risk of rupture of advanced atheromas [4, 14].

Because cardiovascular diseases remain the leading cause of death worldwide, it is necessary to identify new circulating or structural markers for predicting risk for chronic diseases. Studies on the expression of matrix metalloproteinases are important for understanding the biological processes such as cell differentiation and function, related to pathological processes [15], such as coronary artery disease [16]. Some studies suggest that MMP1 gene polymorphisms are associated with enzyme expression levels, including in situ levels (e.g., in atherosclerotic plaques) [17]. For example, MMP1 gene expression may be influenced by a single-nucleotide polymorphism (SNP) in the promoter region of the gene located at the 11q22.3 position, which results in differential MMP1 expression according to the allelic variant [15, 17].

In this context, Chen et al. [18] used the high-throughput, genome-wide scanning procedure that identified an association between the rs495366 SNP of the MMP1 gene and the circulating, serum levels of MMP1. Thus, the study of this polymorphism may help elucidate the pathophysiology of vascular diseases. The general objective of the present study was to test the association between the rs495366 polymorphism of the MMP1 gene with vascular risk factors found in samples of an elderly population of the Brazilian Federal District.

## 2. Methods

### 2.1. Sample

A sample of noninstitutionalized patients aged 60 years or older without clinical symptoms of severe atherosclerosis were recruited between 2011 and 2013 in two general geriatrics outpatient clinics (Geriatric Unit of the Catholic University of Brasília (UCB) and Medical Center for the Elderly of the University of Brasília (UnB)) located in the Metropolitan Region of the Federal District, Brazil. The sample was used to assess the risk for vascular events through a cross-sectional evaluation. Patients recruited at UnB were sent to the Hospital of the Catholic University of Brasília (HUCB) to obtain a standardized baseline evaluation with a clinical protocol conducted by the same medical team.

Inclusion criteria enrolled patients spontaneously seeking primary or secondary care for vascular events at the aforementioned health sites. The exclusion criteria consisted of presenting (i) a clinical history of autoimmune disease or neoplasms of any type, current or past; (ii) report of a pulmonary condition compatible with severe symptomatic obstruction; (iii) severe chronic renal disease determined by creatinine clearance <25 ml·min^−1^; and/or (iv) a clinical history of recurrent infections or chronic inflammation.

The project was approved by the Research Ethics Committee of the School of Medicine of UnB under Protocol number 061/2011. All participants signed an informed consent form.

### 2.2. Clinical and Biochemical Evaluations

The clinical examination consisted of a general evaluation of the patient with measurement of resting blood pressure (BP), cardiac and pulmonary auscultation, superficial and deep palpation of the abdomen, and inspection and digital palpation of the lower limbs according to routine semiological standards. A questionnaire was also administered to obtain information about the socioeconomic profile and medication used, recording the active principles consumed and their classification. Previous circulatory events, such as myocardial infarction or stroke, as well as lifestyle aspects, such as sedentary lifestyle and smoking, were also recorded.

The diagnosis of type 2 diabetes included self-reporting of the condition and fasting glycated hemoglobin (HbA1c) ≥ 6.5%, considering the consumption of hypoglycemic agents or insulin. Individuals were considered to be physically active if they reported 30 minutes or more of exercise for at least four days of the week, whereas smoking was defined as consumption of >100 cigarettes in a lifetime. For the metabolic analysis, the following measurements were obtained according to routine clinical biochemistry and are expressed in standard units: blood glucose, glycated hemoglobin (HbA1c), total cholesterol and fractions, triglycerides, high-sensitivity C-reactive protein, and thyroid-stimulating hormone (TSH). The anthropometric analyses evaluated the body mass index (BMI, in kg/m^2^) and the waist circumference (cm) of each subject.

### 2.3. Measurement of Intima-Media Thickness

The carotid intima-media thickness (cIMT) was determined noninvasively in all participants using a high-resolution ultrasound (Philips, model IE 33) with a 3–9 MHz linear transducer. Measurements of cIMT were performed bilaterally on the distal wall of the common carotid artery bulb and on the origin of the internal carotid artery, using an automated edge-detection software that is capable of displaying the maximum value of 150 measurements of any 10 mm segment [19]. The maximum cIMT was measured manually as the mean of three independent measurements. All clinical procedures and imaging evaluations were performed at the UCB Hospital.

### 2.4. MMP1 Genotyping

Total genomic DNA was obtained using a commercial extraction kit (QIAamp DNA Mini Kit, Qiagen, Brazil). Genotyping reactions were performed using qPCR (real-time PCR) with TaqMan® Universal Master Mix reagent and an assay specific for SNP rs495366 from Thermo Fisher (Massachusetts, USA), with probes designed to detected the flanking regions and the polymorphic variation based on the TTGACCTAAATTTCTGCAAACTATA[R]TCTTATGGTTATGACTCTTTTTGT sequence. The denaturation/extension times and number of cycles used followed the standard program on the equipment used, the QuantStudio 3 Real-Time PCR System (Thermo Fisher®, Massachusetts, USA).

### 2.5. Assessment of Inflammation

At the time of the biochemical evaluations, whole blood was collected. The serum obtained was frozen at −80°C and later thawed for evaluation of immunological mediators. Cytokine concentrations were assessed by traditional immunoenzymatic assays (ELISA) using specific kits manufactured by BD Biosciences (San Diego, CA, USA) used according to the manufacturer's protocols.

Briefly, each serum sample was processed in duplicate together with the standard sample of the mediator provided by the kit, and the readings were made in a BioTek® reader (Winooski, VT, USA). Standard curves for each cytokine were generated by linear regression, and the concentration in each serum sample was determined by interpolation from the corresponding curve.

### 2.6. Statistical Analysis

To test for differences between clinical, biochemical, and anthropometric measures in the sample according to MMP1 genotypes, our statistical analyses compared measures of central tendency by the Mann–Whitney test (continuous variables) or frequencies by the chi-square test. The normal distribution of all variables was assessed using the Kolmogorov–Smirnov test. For these analyses, *P* ≤ 0.05 was adopted as the significance level. All analyses were performed with the Statistical Package for Social Sciences (SPSS) for Windows (version 17.0).

## 3. Results

A total of 366 people aged 60 years and over without clinical symptoms of severe atherosclerosis consecutively treated at the geriatrics units of the participating institutions were included in the study. SNP rs495366 genotyping revealed that homozygotes for the A allele constituted a minority (*n*=30) and were added to the heterozygous group to form a single group (*n*=174) of carriers of the A (A_) allele. The GG genotype group included 192 patients with similar mean age and sex ratio. The genotype proportions did not violate the Hardy–Weinberg equilibrium (*P* > 0.05).

Inferential statistics of the anthropometric, metabolic, and biochemical variables analyzed the individuals according to the genotype groups defined and are presented in Table 1. In purely descriptive terms, the sample was marked by a high prevalence of biochemical and metabolic disorders such as overweight, type 2 diabetes, dyslipidemia, and systemic arterial hypertension, as expected in elderly individuals from a developing country [20]. However, no variations in frequencies or mean values of these findings were found according to MMP1 genotypes.

Similar to the biochemical and metabolic variables, no significant differences between groups were noted for clinical variables except for a sufficient difference in intima-media thickness on the left carotid artery (*P*=0.041) and a trend toward a significance difference on the right counterpart territory (*P*=0.087). Regarding the behavioral variables that increase the risk for vascular events, such as sedentary lifestyle or smoking, no statistically or clinically significant differences were observed.

## 4. Discussion

The main finding of the present study was a significant difference in the intima-media thickness of the left carotid, which was 0.17 mm thicker in carriers of the rs495366 A allele compared with carriers of the GG genotype. The left common carotid artery may be considered more vulnerable to atherosclerosis compared with the right and may be more prone to the development of atherosclerotic plaques and consequently strokes [21, 22] due to greater blood flow in this region, potentiating health problems [23]. Based on the increase in intima wall thickness evidenced here, we can assume that carriers of the A allele of rs495366 SNP may be more prone to stroke, as Lorenz et al. [24] demonstrated that an increase in carotid thickness of 0.10 mm increases the risk for stroke by 17%.

Several genetic factors are considered determinants for the development of atherosclerotic disease. Approximately 400 genes may be involved in the regulation of various factors, such as endothelial function, coagulation, inflammation, and lipid metabolism [25]. Among these, we highlight the MMP1 (collagenase-1) polymorphism given that an imbalance in its activity is reported in the literature as a factor that contributes to plaque instability in atheromas [26].

Increased MMP1 expression in the early stages of an atherosclerotic process may be a compensatory response that involves tissue remodeling under physiological stress to delay the progression to thromboembolic events [26]. However, one cannot ignore the hypothesis that MMP1 plays a pathophysiological role via excessive accumulation as a result of smooth muscle cell accumulation in a necrotic core under a fibrous cap where it weakens high-stress regions [6], predisposing the plaque to rupture [4, 27]. Despite this hypothesis, MMP1 activation is essential for vascular wall remodeling to prevent excessive growth of the intima layer in response to lesions mainly by migration or reprogramming of vascular smooth muscle cells. In this sense, it is plausible that the observed association arises from an impaired ability to promote remodeling among carriers of the A allele, which is associated with reduced enzyme expression in the tissues of carriers of this allelic form [6]. Galon et al. [16] suggested that lower MMP1 expression leads to the accumulation of extracellular matrix proteins, contributing to the early formation of vascular lesions and structural changes that promote rupture of atheromatous plaques.

The protective role of enhanced MMP1 expression is supported by Lemaître et al. [28] and by Ye et al. [26], suggesting that increased MMP1 expression during atherogenesis results in smaller and less severe lesions with a lower collagen content, which is compatible with negative remodeling (degradation of the collagen matrix) that delays the progression of atherogenesis and results in more stable lesions.

Regarding the validity of our findings, assuming that both genotypic groups were approximately equal in size, the equation by Whitley and Ball [29] was used to calculate a posteriori the power of our study to detect a change in carotid thickness. Taking into account the left carotid measurements where the difference in mean values between genotypes was 0.17 mm and the general standard deviation of the variable was 0.62 mm, the standardized difference to be detected was 0.17/0.62 = 0.274 (0.3). Thus, the number of participants needed to detect a difference with a discriminatory power of 80% and a 5% statistical significance level was calculated as 176 subjects in each group. Our study was very close to the sample size needed to consider it representative, as it was only short two individuals in the group of G homozygotes.

It should be recognized that the onset of atherosclerotic plaques is multifactorial mostly due to unhealthy lifestyle habits, such as a diet rich in lipids and carbohydrates, a sedentary lifestyle, and smoking. Although it was not possible to investigate the dietary intake of the patients, our study contributes to the literature by demonstrating that the latter aspects were similar between groups and thus did not significantly influence the conclusions presented. Similarly, and consistent with several studies that analyzed the impact of different risk factors for stroke in several populations worldwide [30–34], the frequencies of classic risk factors for stroke (namely, hypertension, dyslipidemias, obesity, and diabetes) did not differ between genotype groups, possibly revealing an allelic effect that outweighs the classic determinants when homogeneous between groups. Even systemic inflammatory factors, which are associated with lateral inequality between carotid branches [35], do not seem to have an effect in our recent findings. And despite the lack of an actual assessment on MMP1 levels, the authors consider that such limitation was addressed and, to some extent, lessened by an investigation that relied precisely on a genetic variation with solid evidence for an effect on MMP1 levels. Therefore, our conditions considered the best possible proxy.

However, it is important to recognize that matrix degradation is determined not only by the tissue expression of metalloproteinases but also by the activation pattern of the enzymes in the extracellular space, the degree of inhibition promoted by endogenous inhibitors, and the presence and activity of enzymes that do not directly degrade the metalloprotease matrix. Thus, a better understanding of the elements involved and the dynamics of matrix degradation in the fibrous plaque in high-stress regions is needed to provide information on the progression of stable and unstable plaques in the circulatory tree [6].

## 5. Conclusion

In this study, subjects carrying the allele associated with lower MMP1 expression exhibited increased left carotid intima-media thickness, and this finding is presumed to increase the risk of stroke among elderly outpatients. When imbalanced, MMP1 activity in advanced atheroma may be one of the contributing factors of plaque instability. Thus, we assume that increased MMP1 expression in the early stages of atherogenesis may be a beneficial adaptive response.

## Figures and Tables

**Table 1:** Anthropometric, clinical, metabolic, and inflammatory variables of the sample according to the genotypic group.

Variables	rs495366 genotypes		*P*
	GG (*n*=174)	A_ (*n*=192)	
Age (years)	79.5 ± 9.3	79.2 ± 8.3	0.745
Male (%)	32.2	28.1	0.398
BMI (kg·m^−2^)	26.9 ± 4.9	26.9 ± 5.1	0.841
WC (cm)	96.3 ± 12.4	94.7 ± 11.4	0.169
SBP (mm Hg)	138.8 ± 20.4	142.0 ± 21.2	0.150
DBP (mm Hg)	78.1 ± 11.0	77.2 ± 11.5	0.268
SAH (%)	76.4	76.0	0.929
Glucose level (mg·dl^−1^)	101.8 ± 22.6	105.4 ± 43.0	0.659
HbA1c (%)	6.0 ± 0.9	6.1 ± 1.3	0.365
DM2 (%)	25.3	21.4	0.374
Total cholesterol (mg·dl^−1^)	194.4 ± 40.0	203.2 ± 84.2	0.297
Triglycerides (mg·dl^−1^)	131.5 ± 58.8	135.3 ± 62.6	0.498
HDL-c (mg·dl^−1^)	51.7 ± 12.9	51.5 ± 13.2	0.988
LDL-c (mg·dl^−1^)	113.2 ± 34.1	115.9 ± 36.2	0.407
VLDL-c (mg·dl^−1^)	29.3 ± 13.4	30.0 ± 13.9	0.482
Creatinine clearance (ml·min^−1^)	60.3 ± 26.4	55.6 ± 24.1	0.120
CRP (mg·dl^−1^)	1.61 [2.25]	1.70 [3.10]	0.636
TSH (mIU·l^−1^)	2.16 [2.05]	2.15 [2.53]	0.976
TNF	0.00 [0.60]	0.00 [0.34]	0.684
IL12	0.00 [8.92]	0.00 [8.93]	0.551
IL10	0.84 [2.84]	1.24 [2.94]	0.599
Sedentary (%)	59.2	63.0	0.453
Smoker (%)	19.5	17.2	0.561
Right cIMT (mm)	1.28 ± 0.60	1.40 ± 0.66	0.087
Left cIMT (mm)	1.27 ± 0.60	1.44 ± 0.64	0.041

Data are expressed as average ± standard deviation for normally distributed continuous parameters, relative frequencies within genotype for categorical features, and median with the interquartile range in brackets for nonnormally distributed continuous variables. BMI = body mass index; WC = waist circumference; HbA1c = glycated hemoglobin A1c; DM2 = type 2 diabetes mellitus; VLDL-c = very low-density lipoprotein cholesterol; LDL-c = low-density lipoprotein cholesterol; HDL-c = high-density lipoprotein cholesterol; SBP = systolic blood pressure; DBP = diastolic blood pressure; SAH = systemic arterial hypertension; CRP = C-reactive protein; TSH = thyroid-stimulating hormone; cIMT = carotid intima-media thickness.

## Data Availability

The datasets generated during and/or analyzed during the current study are available from the corresponding author on reasonable request.
